# Immunology of membranous nephropathy

**DOI:** 10.12688/f1000research.17589.1

**Published:** 2019-05-24

**Authors:** Shin'ichi Akiyama, Enyu Imai, Shoichi Maruyama

**Affiliations:** 1 Division of Nephrology, Department of Internal Medicine, Nagoya University Graduate School of Medicine, Nagoya, Japan; 2 Nakayamadera Imai Clinic, Takarazuka, Hyōgo, Japan

**Keywords:** NEP, PLA2R, THSD7A, Epitope spreading, Heymann nephritis, Membranous nephropathy, podocyte

## Abstract

Accounting for about 20 to 50% of cases of primary nephrotic syndrome, membranous nephropathy (MN) is the leading cause of nephrotic syndrome in adults. A rat model created nearly 60 years ago to research the primary MN disorder, Heymann nephritis, has provided us with a plethora of important information. Recently, our knowledge about MN has dramatically progressed. Heymann nephritis and human MN are now known to share a high degree of similarity in pathogenesis. This review summarizes our current understanding of MN pathogenesis while focusing particularly on the immunological aspects.

## Introduction

Membranous nephropathy (MN) is an organ-specific autoimmune disease and a leading cause of nephrotic syndrome (NS) in adults. It is classified as either primary or secondary MN depending on its etiology. Secondary MN can be caused by cancers, infections, autoimmune diseases such as systemic erythematosus, or drugs. Overall, 30 to 40% of patients with MN develop end-stage kidney disease within 5 to 15 years of onset ^1^. Moreover, mortality from MN is high because of complications such as infections, cardiovascular events, or malignancies ^2^.

Renal biopsy is used to diagnose MN. Light microscopy of periodic acid methenamine silver (PAM)-stained kidney sections shows a bubbling appearance and spike formation of the glomerular basement membrane (GBM) because of its expansion between and around immune deposits. Furthermore, immunostaining with IgG (especially IgG4) and C3 shows a granular pattern along the GBM. Electronic microscopy shows subepithelial electron-dense deposits. These findings indicate that autoimmunity takes place during MN development. However, the precise pathogenesis of human MN has long remained unclear.

The history of MN research started with the construction of an animal model nearly 60 years ago. Heymann *et al*. ^3^ reported that injecting a fraction of the renal brush border membrane induced MN in rats. Since then, numerous researchers have studied this model and many hypotheses on the pathogenesis of MN have been proposed from their studies. However, only recently were these hypotheses proven in human MN. The study of MN is one of the most successful examples of translational science in kidney research. In this review article, we discuss the history of MN research and describe new insights regarding human MN pathogenesis from the past decade.

## Pivotal breakthroughs in the history of membranous nephropathy research

### Heymann nephritis

Current concepts regarding MN pathogenesis are derived largely from early studies carried out on the Heymann nephritis model ^3^. Heymann *et al*. injected crude kidney extracts combined with Freund’s adjuvant in rats in order to develop NS ^3^. The authors then showed that the tubular extracts, and not glomerular extracts, induced nephropathy. Notably, the rats developed autoantibodies against tubular extracts, which induced MN. This model is called the active Heymann nephritis model.

Subsequently, the passive Heymann nephritis (PHN) model was designed ^4^. The insoluble sub-fraction from the brush borders of rat proximal tubules, termed fraction 1A (Fx1A), was isolated and injected into sheep to produce antibodies (Abs) that caused severe proteinuria when injected into rats. These rats that were administered anti-Fx1A Abs also developed subepithelial deposits, which were visible when stained with IgG, C3, and C5b-9 ^5^. A key finding showed that the immune deposits formed *in situ* as a result of the Abs binding to an intrinsic glomerular antigen.

GP330, also known as megalin, was one of the major proteins found within the Fx1A. Further studies revealed that megalin was expressed on the brush borders of proximal tubular cells and cell surfaces of podocytes. Abs against Fx1A bound to megalin on podocyte membranes and formed immune complexes *in situ* that led to complement activation. Furthermore, the membrane attack complex, C5b-9, damaged podocytes and induced slit diaphragm dysfunction, thereby leading to protein leakage from glomeruli ^5^. Epitope spreading in a megalin-induced active Heymann model was reported by Shah *et al*. in 2007 ^6^.

The findings from studies on PHN provide us with several hypotheses, which may also apply to human MN. The main hypotheses are as follows: (1) the antigenic protein in human MN expresses itself on the cell membrane of podocytes; (2) the Abs bind to the target protein at the base of the podocyte membrane, thereby forming immune deposits *in situ*; (3) immune complexes induce complement activation, resulting in podocyte injury; and (4) target epitopes change as the disease progresses.

### Neutral endopeptidase

Abs against podocyte antigens that induce MN in humans were first confirmed in 2002, when Debiec *et al*. ^7^ presented a case of neonatal NS induced by alloimmune Abs against NEP (neutral endopeptidase or membrane metalloendopeptidase; National Center for Biotechnology Information [NCBI] gene ID 4311). The authors found that the mother had truncating mutations in exons 7 and 15 (compound heterozygote) of the NEP gene, lacked NEP expression, and developed anti-NEP Abs during a previous pregnancy. The NEP Abs were transferred from the NEP-deficient mother to her infant, thus inducing severe NS, which was resolved 11 months after birth. The authors also showed that NEP was expressed on the cell surface of podocytes and that the maternal anti-NEP Abs passed through the GMB, reached the basal membrane of podocytes, and formed immune complexes *in situ*, exhibiting NEP expression on the podocytes of the infant ^8^. The mechanisms involved here have many things in common with those proposed by the studies on PHN.

### PLA2R and THSD7A

**Figure 1.**
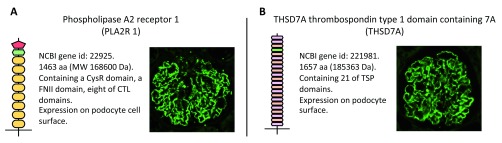
PLA2R and THSD7A protein structures and their staining images in the glomeruli of a patient with membranous nephropathy (MN). PLA2R ( **A**) and THSD7A ( **B**) are corresponding antigens of primary MN. These two proteins have similar structures. Autoantibodies against PLA2R and THSD7A in patients with MN can bind to epitopes only under non-reducing conditions. They show enhanced granular expression on podocyte surfaces in patients with MN after indirect immunofluorescence staining with Abs against PLA2R or THSD7A. CTL, C-type lectin; MW, molecular weight; NCBI, National Center for Biotechnology Information; PLA2R, phospholipase A2 receptor; THSD7A, thrombospondin type 1 domain-containing 7A; TSP, thrombospondin.

In 2009, Beck claimed that he found a target autoantigen in human MN ^9^. He showed that 70% of adult patients with primary MN (pMN) ^9^ exhibited IgG4 Abs against the M-type phospholipase A2 receptor (PLA2R; NCBI gene ID 22925; Figure 1A) expressed in podocytes; however, no patients with secondary MN were positive for the same. The Abs were detected in the serum and in the deposits on the GBM of glomeruli ^10^. Many other laboratories reported the prevalence of PLA2R Abs in MN patients were 70 to 85% ^10– 14^.

In 2014, Tomas *et al*. ^15^ reported a second IgG4 autoantibody specific for thrombospondin type 1 domain-containing 7A (THSD7A; NCBI gene ID 221981; Figure 1B), which was another podocyte membrane antigen. THSD7A exhibited properties that were similar to those of PLA2R. The dominant subclass of autoantibodies is IgG4, which specifically recognizes the conformational epitopes on the membrane protein expressed on podocytes. THSD7A Abs were identified in a smaller percentage of patients with pMN (2–5%) ^14, 15^. Even though the existence of dual Abs against both PLA2R and THSD7A has been reported, these cases are rare ^9^. Therefore, the autoantibodies were detected in 75 to 90% of total patients with pMN. Notably, the pathological Ab remains to be elucidated in only a small proportion of patients with MN.

Both proteins are multi-domain transmembrane glycoproteins composed of multiple repeating domains, the structure of which depends on several disulfide bonds per domain. Notably, PLA2R and THSD7A Abs recognize steric epitopes that appear on the surface of several domains. The pathological role of anti-THSD7A Abs was established in mice that developed MN features after the adoptive transfer of human anti-THSD7A Abs.

## Features of autoantibodies and clinical applications as biomarkers

### Diagnostic value of autoantibodies

One of the special properties of PLA2R and THSD7A Abs is their high specificity. Previous reports have demonstrated that some number of secondary MN patients experiencing sarcoidosis, hepatitis B, other virus infections and cancer, and lupus nephritis were positive for PLA2R Abs ^16, 17^. However, these cases can be chalked up to the coexistence of secondary diseases with PLA2R- or THSD7A-related MN. What is important is the fact that there has never been a positive PLA2R or THSD7A Ab patient who was not diagnosed with MN. In other words, PLA2R or THSD7A Ab is 100% specific in terms of MN diagnosis. ****

These two Abs are excellent biomarkers for diagnosing MN. PLA2R Abs are more prevalent among old males, whereas THSD7A Abs are more prevalent in relatively young females. Unfortunately, clinical information is not always clear to distinguish primary from secondary MN cases. However, PLA2R and THSD7A Abs have great clinical significance as biomarkers because the presence or absence of these biomarkers not only facilitates MN diagnosis in patients with NS but also helps classify patients with MN as primary or secondary MN cases. Moreover, measuring PLA2R Ab levels helps identify patients with poor renal prognosis because a number of studies have reported that the Ab titer correlates with treatment failure and future kidney dysfunction.

### Temporal change in autoantibody-driven pathogenesis in PLA2R-associated membranous nephropathy

**Figure 2.**
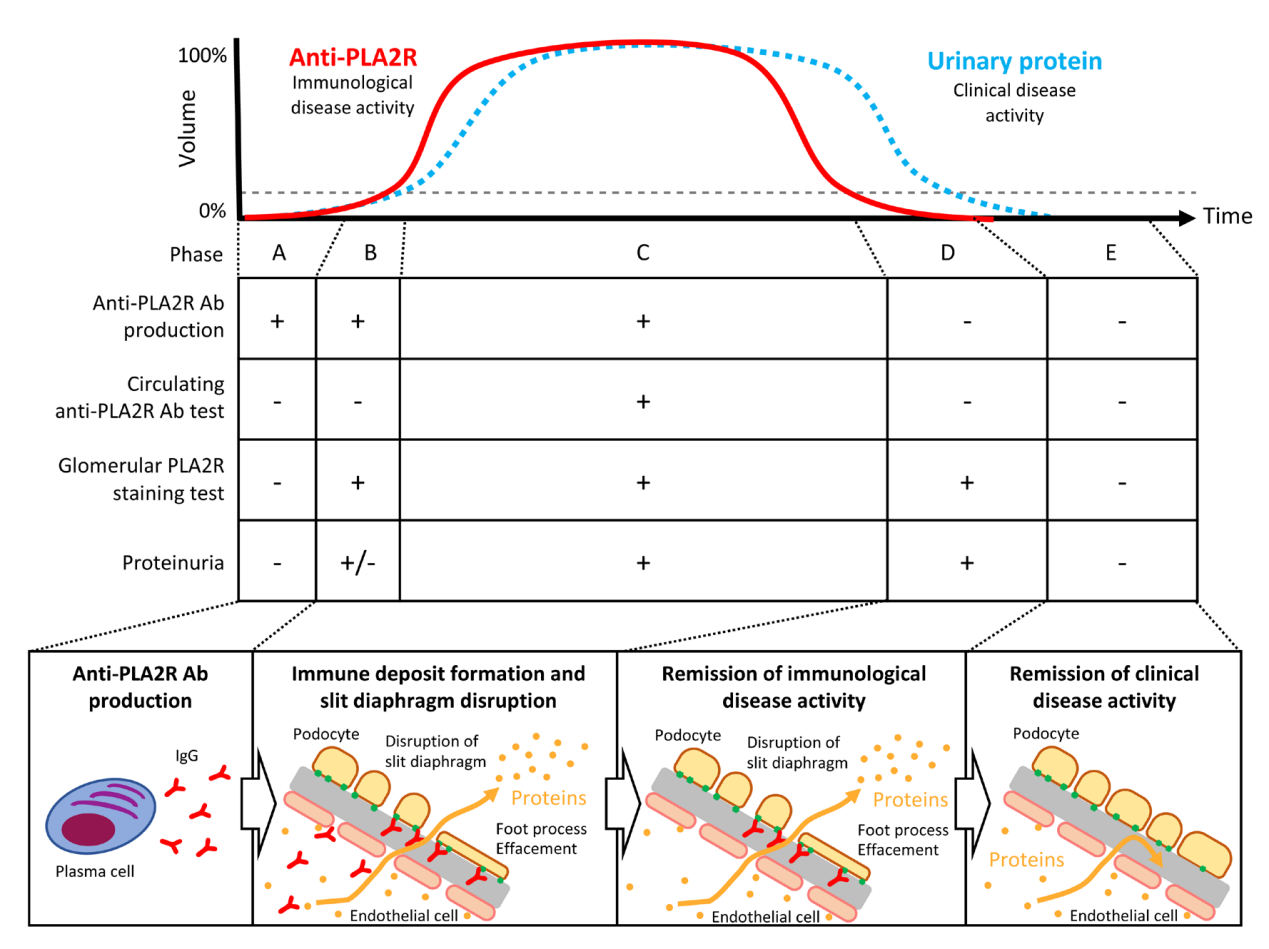
Schematic of autoantibody-driven temporal pathogenesis in PLA2R-associated membranous nephropathy. The graph shows the temporal change in circulating anti-PLA2R antibodies (Abs) and urinary protein levels (modified from Francis *et al*. ^26^). The middle table shows the disease phases classified on the basis of Ab production, serum tests for Abs in circulation, glomerular staining for PLA2R, and proteinuria. The bottom schema illustrates the course of the disease from Ab production to proteinuria remission. PLA2R, phospholipase A2 receptor.

After plasma cells are initiated to produce Abs against PLA2R (phase A, Figure 2), the Abs immediately bind to podocytes and proteinuria begins to develop. In this phase, no Abs or only a low level of Abs is detected in circulation (phase B, Figure 2). This phenomenon is known as the “kidney as a sink” mechanism ^18^. Eventually, proteinuria starts to increase and Abs are detected in the serum, the timing of which corresponds to a clinically active phase (phase C, Figure 2). When the disease starts to resolve, Ab production stops and disappears from circulation but not from the glomeruli (phase D, Figure 2). Finally, podocyte injury is restored and no proteinuria is detected during the remission phase (phase E, Figure 2).

### Intermolecular epitope spreading of anti-PLA2R antibodies

**Figure 3.**
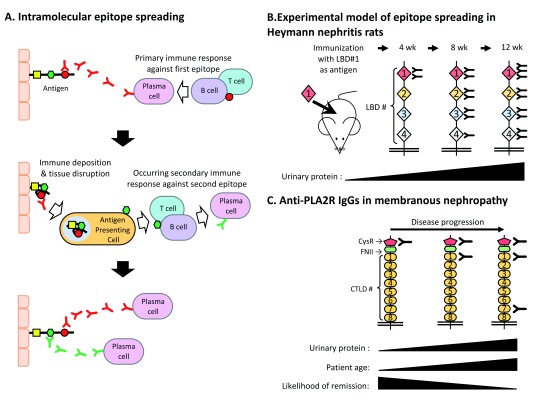
Schematic of epitope spreading. ( **A**) Intramolecular epitope spreading. The upper panel shows the primary immune response to the outermost epitope of the target antigen. The middle panel shows the secondary immune response to the inner epitopes caused by tissue destruction and presentation of the inner epitopes. The lower panel shows the result of epitope spreading. ( **B**) Experimental model of Heymann nephritis–induced epitope spreading in rats. The outermost megalin epitope (LBD#1) was injected into rats, which initiated antibody (Ab) production against inner epitopes (LBD#2–LBD#4), eventually leading to severe proteinuria. ( **C**) Clinical evidence of epitope spreading in human membranous nephropathy (MN). In MN patients with PLA2R Abs, the target epitope changes from the outermost epitope (CysR) to the inner epitopes (CTDL1 to CTDL7) as the disease progresses. Patients exhibiting Abs against the inner epitopes tend to be old and resistant to therapy. PLA2R, phospholipase A2 receptor.

Epitope spreading is a phenomenon involving epitope diversification that is recognized by the immune system ^19^ ( Figure 3A). Intramolecular epitope spreading has already been reported in patients with autoimmune diseases such as systemic erythematosus ^20, 21^, Sjögren’s syndrome ^22^, rheumatoid arthritis ^23^, scleroderma ^24^, and type 1 diabetes mellitus ^25^. Primarily, autoimmune responses begin reacting with the outermost epitope of the target antigen. As tissue damage progresses, antigen-presenting cells present inner epitopes, thus resulting in secondary responses.

Experimental evidence for epitope spreading in experimental MN was previously reported by Shah *et al*. ^6^ ( Figure 3B). They produced a recombinant LBD#1, which was the outermost epitope of megalin. They injected this epitope into rats, and NS (active Heymann nephritis) was observed to develop successfully. As proteinuria increased, the reactive epitope progressed from LBD#2 to LBD#4.

Furthermore, clinical evidence for epitope spreading in human MN was reported by Seitz-Polski *et al*. ^27^ ( Figure 3C). During the early phase of MN that contained positive PLA2R Abs, CysR (the outermost epitope) was used as the target epitope. As the disease progressed, Abs against inner epitopes (that is, CTDL1 against CTDL7) were produced. The authors also showed that patients who had Abs against inner epitopes tended to be old and more resistant to therapy. Therefore, analysis of PLA2R1 epitope spreading is a powerful tool in terms of monitoring disease phase and severity and predicting the renal prognosis of patients with MN.

## Immunology of PLA2R and THSD7A antibodies

Despite numerous reports on these two autoantibodies in patients with MN, their actual immunological processes remain largely unknown. First, a loss of tolerance for podocyte-expressed antigens occurs in patients with MN. The number of patients with MN has reportedly increased in China, especially in urban cities, and air pollution—as caused, for example, by particulate matter (PM) 2.5—may be related to this increase ^28^. It is possible that the immunological activation induced by environmental factors leads to the loss of B-cell tolerance. Second, IgG4 Ab generation and deposition on GBM are observed. IgG4, which does not have the ability to strongly activate complements, has been shown to be the predominant subclass in PLA2R and THSD7A Abs. Notably, Debiec *et al.* ^29^ reported a post-kidney transplant case in which the generation of IgG3κ in PLA2R Abs led to recurrent MN. Therefore, the role of IgG4 remains to be clarified. Lastly, complement activation is demonstrated to play a crucial role in MN, at least in animal models ^30– 32^. However, a clinical study using the anti-C5 Ab, eculizumab, against patients with MN failed to show significant effects ^33^. In contrast, recent studies successfully showed that the anti-CD20 Ab, rituximab, depleted B cells, which led to proteinuria remission in patients with MN. Although the precise mechanisms are not known, there is no doubt that B cells play a crucial role in MN.

## Regional difference

Recently, MN research has provided us with clinically useful information, especially with respect to target antigens and corresponding Abs. Although the reported rates varied between countries, the prevalences of PLA2R-associated MN, THSD7A-associated MN, and pMN without any known Abs were reported to be 80–85%, 3–5%, and 10–15%, respectively ^9^. Additionally, we reported that the prevalence of anti-PLA2R Abs in Japanese patients with pMN was about 50%, which was lower than in other countries. The overall prevalences of the three types of MN in Japan were 50–70%, 3–10%, and 20–45%, respectively ^34– 37^. There are several possible reasons for this discrepancy between Japan and other countries. First, genetic differences likely exist between patients in Japan and those in other countries. However, it may be difficult to explain the differences in genetic background between Japanese, Korean, and Chinese cohorts. Second, unknown environmental or dietary factors may affect the results. As mentioned above, PM 2.5 may have increased the number of MN patients in China ^28^. Lastly, discrepancy can be attributed to health check systems and their ability to help identify individuals with low levels of urinary protein; that is, in Japanese patients, MN may be treated at a very early stage before PLA2R Abs are detectable in the circulation. However, these hypotheses need to be examined in future studies. Unraveling global differences in the prevalence of these three types of pMN can provide clues to fundamentally improve our understanding of pMN pathogenesis.

## Conclusions

Our knowledge about pMN has remarkably progressed over the past few decades. Hypotheses of human MN pathogenesis were formulated from studies based on the first MN (Heymann nephritis) animal model. It is of great interest that these hypotheses have recently been proven in human studies. However, the fundamental pathophysiological mechanism of MN remains largely unknown. It is important to note that MN research has not reached its end. There is still much to accomplish before we fully understand MN immunology.
